# Supplementary material for: Content-rich biological network constructed by mining PubMed abstracts
Source: BMC Bioinformatics. 2004 Oct 8;5:147. doi: 10.1186/1471-2105-5-147 (PMC528731; doi:10.1186/1471-2105-5-147)
Supplement: Additional File 2 — The original results of the above study (non-essential files are deleted to keep the file size under the limit set by BMC bioinformatics). [file 1471-2105-5-147-S2.bz2 › chilibotAdditionalFile2/dip20/203ID7664335E693/html/ERCC6_CKN1.html]

 


 **ERCC6** and **CKN1** 
  
Found 57 abstracts in PubMed, retrieved 20.  
 

 What does Google say? 
 PDF only 
| .edu only 

---

**Interactive relationship** (e.g. stimulation, inhibition, etc)

**Neutral relationship**- The hydrogen peroxide induced translocation of CSA  [ **CKN1** ]  was also CSB  [ **ERCC6** ]  dependent.  Ref: 11782547 Proc Natl Acad Sci U S A, 2002
- The translocation of CSA  [ **CKN1** ]  was independent of Xeroderma pigmentosum group C, which is specific to the global genome repair subpathway of nucleotide excision repair and of the core NER factor Xeroderma pigmentosum group A but required the CSB  [ **ERCC6** ]  protein.  Ref: 11782547 Proc Natl Acad Sci U S A, 2002

**Non-interactive relationship** (e.g. studied together, co-existance, homology, etc.)

- Cellulose acetate electrophoresis analysis revealed that RPC C2A cells synthesized CSA  [ **CKN1** ]  and CSB  [ **ERCC6** ]  but not CSC.  Ref: 11199713 J Endod, 2000
- Two genes are involved CSA  [ **CKN1** ]  and CSB  [ **ERCC6** ] .  Ref: 12560492 Nucleic Acids Res, 2003
- 10 ng ml of TGF beta increased the production of CSA  [ **CKN1** ]  and CSB  [ **ERCC6** ]  in the cell ECM fraction.  Ref: 11199713 J Endod, 2000
- Deletion of the gene encoding the Pol II elongation factor TFIIS resulted in enhanced UV sensitivity, but only in the absence of global genome repair dependent on the RAD7 and RAD16 genes, a result seen previously with deletions of RAD26  [ **ERCC6** ]  and RAD28, yeast homologs of the human Cockayne syndrome genes CSB  [ **ERCC6** ]  and CSA  [ **CKN1** ] , respectively.  Ref: 11254132 Mol Gen Genet, 2001
- Designated CsA  [ **CKN1** ]  and CsB  [ **ERCC6** ] , their homokaryotic nature was established by polymerase chain reaction amplification and sequence analysis of the allelic variants of three MnP genes.  Ref: 11356573 FEMS Microbiol Lett, 2001
- The abnormalities in CS are associated with mutations in the CSA  [ **CKN1** ]  or CSB  [ **ERCC6** ]  genes.  Ref: 10739753 Am J Hum Genet, 2000
- The disease is caused by the disruption of CSA  [ **CKN1** ] , CSB  [ **ERCC6** ] , or some types of xeroderma pigmentosum genes.  Ref: 12665480 FASEB J, 2003
- Traditionally, CSA  [ **CKN1** ]  and CSB  [ **ERCC6** ]  have been ascribed a function in the transcription coupled repair pathway of nucleotide excision repair that efficiently removes bulky lesions from the transcribed strand of RNA polymerase II transcribed genes.  Ref: 12032859 Oncogene, 2002
- Despite this DNA repair defect, individuals with CS group A CSA  [ **CKN1** ]  or group B CSB  [ **ERCC6** ]  do not exhibit an increased spontaneous or UV induced cancer rate.  Ref: 11238917 Mol Cell Biol, 2001
- ... survival, repair and recovery from transcription inhibition critically depend on CSA  [ **CKN1** ] , CSB  [ **ERCC6** ]  and UVS, the factors specific for transcription coupled NER.  Ref: 12531012 DNA Repair Amst , 2002
- Cockayne syndrome CS is a segmental premature aging syndrome in humans that has two complementation groups, CSA  [ **CKN1** ]  and CSB  [ **ERCC6** ] .  Ref: 12483520 Oncogene, 2002
- Moreover, studies of cells deficient in XPC or in the genes implicated in Cockayne syndrome CSA  [ **CKN1** ]  and CSB  [ **ERCC6** ]  indicated that the drug sensitivity is specifically dependent on the transcription coupled pathway of NER.  Ref: 11479630 Nat Med, 2001
- These findings indicate that CSA  [ **CKN1** ]  and CSB  [ **ERCC6** ]  are major sulfated GAGs synthesized by RPC C2A cells.  Ref: 11199713 J Endod, 2000
- In addition to interacting with XPA, immunoprecipitation experiments demonstrated that a fraction of XAB2 is able to interact with the transcription coupled repair specific proteins CSA  [ **CKN1** ]  and CSB  [ **ERCC6** ]  as well as RNA polymerase II.  Ref: 10944529 J Biol Chem, 2000
- The chondroitin sulfates CSA  [ **CKN1** ] , CSB  [ **ERCC6** ] , CSC had far less impact upon APTT with the FXa FXDP system.  Ref: 12430983 Can J Physiol Pharmacol, 2002
- Despite this DNA repair defect, individuals with CS of which there are two complementation groups, CSA  [ **CKN1** ]  and CSB  [ **ERCC6** ]  do not demonstrate an elevated incidence of cancer.  Ref: 11713576 Int J Oncol, 2001
- The UV induced degradation rates of RNAP II LS were not significantly altered when comparing CSA  [ **CKN1** ]  or CSB  [ **ERCC6** ]  to repair competent control cells.  Ref: 11516929 Mutat Res, 2001
- Dental pulps contain sulfated glycosaminoglycans GAGs, such as chondroitin 4 sulfate CSA  [ **CKN1** ]  4CS, dermatan sulfate CSB  [ **ERCC6** ]  DS, and chondroitin 6 sulfate CSC 6CS.  Ref: 11199713 J Endod, 2000
- The abnormalities in CS are caused by mutated CSA  [ **CKN1** ]  or CSB  [ **ERCC6** ]  genes.  Ref: 11443545 Am J Hum Genet, 2001
- Measurement of 35S sulfate incorporation showed a significant increase in the amount of GAGs by TGF beta, 1.3 fold CSA  [ **CKN1** ] , and 1.2 fold CSB  [ **ERCC6** ]  in the cell ECM fraction.  Ref: 11199713 J Endod, 2000
- Defects in nucleotide excision repair NER as defined by the UV sensitivity of xeroderma pigmentosum XP, Cockayne syndrome CS and trichothiodystrophy TTD patients has lead to the identification of most of the genes involved XPA through XPG, CSA  [ **CKN1** ]  and CSB  [ **ERCC6** ] .  Ref: 11104904 Mutat Res, 2001
- Comparison of the activities of CSA  [ **CKN1** ]  and CSB  [ **ERCC6** ]  oligo and polysaccharides with a similar sulfation pattern and content achieved from partial desulfation demonstrated that glucuronic acid rather than iduronic acid residues are important for IRBC binding.  Ref: 11956186 J Biol Chem, 2002
